# Can we differentiate minimally invasive adenocarcinoma and non-invasive neoplasms based on high-resolution computed tomography features of pure ground glass nodules?

**DOI:** 10.1371/journal.pone.0180502

**Published:** 2017-07-06

**Authors:** Xiaoye Wang, Lihua Wang, Weisheng Zhang, Hong Zhao, Feng Li

**Affiliations:** 1 Department of Radiology, First Affiliated Hospital of Dalian Medical University, Dalian, China; 2 Department of Radiology, Second People’s Hospital of Zhuhai, Zhuhai, China; 3 Medical Imaging Department, Qingdao Women and Children’s Hospital, Qingdao, China; Peking University People's Hospital, CHINA

## Abstract

**Objective:**

The purpose of our study was to assess the differentially diagnostic value of radiographic characteristics of pure ground glass nodules (GGNs) between minimally invasive adenocarcinoma and non-invasive neoplasm.

**Methods:**

Sixty-seven pure GGNs (28 minimally invasive adenocarcinomas (MIA) and 39 pre-invasive lesions) were analyzed from June 2012 to June 2015. Pre-invasive lesions consisted of 15 atypical adenomatous hyperplasia (AAH) and 24 adenocarcinomas in situ (AIS). High-resolution computed tomography (HRCT) features and volume of MIA and pre-invasive lesions were assessed. Fisher exact test, independent sample *t* test, Mann-Whitney U test and receiver operating characteristic (ROC) curve analysis were performed.

**Results:**

Inter-observer agreement indexes for the diameter, mean HRCT attenuations and volume of pure GGNs were all high (ICC>0.75). Univariate analyses showed that lesion diameter, mean HRCT attenuation, and volume value differed significantly between two groups. Among HRCT findings, GGN shape as round or oval (F = 13.456, P = 0.002) and lesion borders as smooth or notched (F = 15.742, P = 0.001) frequently appeared in pre-invasive lesions in comparison with MIA. Type II and type III of the relationship between blood vessels and pure GGNs suggested higher possibility of malignancy than type I.

**Conclusions:**

HRCT features of pure GGNs can help to differentiate MIA from non-invasive neoplasms.

## Introduction

Pure ground-glass nodules (GGN) are defined as focal nodular areas of increased lung attenuation on high-resolution computed tomography (HRCT), in which the pulmonary vessels and bronchia structures through the nodule can be observed [1]. Most of persistent pure GGNs are diagnosed as focal interstitial fibrosis, adenomatous hyperplasia (AAH), adenocarcinoma in situ (AIS), minimally invasive adenocarcinoma (MIA), or even invasive adenocarcinoma [2, 3].Previous studies have classified the treatment of pure GGNs into two groups: one for appropriate treatment and the other for following-up [4]_._Which kind of pure GGNs should be resected or followed up? Can we predict the histologic subtypes and prognosis of pure GGNs? CT has been implemented on regular health check-up and HRCT enables these nodules to be investigated in more details, which are helpful for detecting malignant tumors at earlier stage. Most pure GGNs are indolent and often diagnosed as pre-invasive adenocarcinoma or MIA [5].

Malignancy rate of pure GGNs has been reported ranging from 18% to 48% [6, 7]. The rate of 5-year disease-free survival of low grade adenocarcinoma has been evaluated as 100% of AIS and near 100% disease-specific survival of MIA [8]. Thus, differential diagnosis of pre-invasive adenocarcinoma and MIA before surgery is important and it can help clinicians to determine appropriate managing strategies for pure-GGNs, which one should be resected or which one can be followed-up according to imaging findings. Pre-invasive lesions are significantly smaller and more frequently non-lobulated than invasive adenocarcinoma in pure GGNs [9]. The initial mean CT attenuation and tumor size of ≥7 mm of pure GGNs are risk factors for tumor growth [10]. The sphericity is seen more frequently with AAH and the presence of internal air bronchograms is more often seen in adenocarcinoma according to a report [11]. Several investigations reported that the notched signs, spiculations, bubbly lucencies, and rapid volume expansion are more common in cases of invasive adenocarcinomas [12–14]. The diagnostic value of radiographic characteristics of GGN has become a major focus. However, the result of above previous studies was notable to differentiate pre-invasive lesions (AAH and AIS) from MIA when they appear as pure GGNs at HRCT. We hypothesized that HRCT imaging and image-data processing techniques would allow us to detect the characteristics of pre-invasive adenocarcinoma and MIA, which appear as small and pure GGNs.

Therefore, we aimed to analyze HRCT features of pure GGNs pathologically diagnosed as MIA and pre-invasive adenocarcinoma for patient stratification and different treatment in clinic.

## Materials and methods

### Subjects

This retrospective study was approved by the institutional review board of First Affiliated Hospital of Dalian Medical University.

Sixty-seven pure GGNs of 67 Patients (13 men and 54 women; age range, 17–79 years; mean age ± standard deviation, 55.8 years ± 1.4) from June 2012 to June 2015 were selected retrospectively. All patients underwent a preoperative HRCT examination.

The lesions of size between 5mm and 30mmwere included while the others of size less than 5mm or greater than 30mm were excluded. Fifteen lesions were diagnosed as AAH, 24 as AIS, and 28 as MIA. The differential diagnosis of AAH, AIS and MIA were based on novel classification criteria (15)proposed by International Association for the Study of Lung Cancer(IASLC), the American Thoracic Society (ATS), and the European Respiratory Society (ERS) in 2011. Pre-invasive lesions included AAH and AIS, which were defined as lesions showing no stromal, vascular, or pleural invasion [15]. MIA was defined as a predominantly lepidic lesion lacking of necrosis and any invasion of lymphatics, blood vessels, or pleura and measuring 30 mm or smaller [15] (Table 1).

**Table 1:** Characteristics of pure GGNs (n = 67).

Characteristics	Frequency
**Sex, n (%)**	
Male	13 (19.4)
Female	54 (80.6)
**Age, mean (range), years**	**55.81 (17–19)**
**Location, n (%)**	
RUL/LUL	28 (41.8) / 18 (26.9)
RML	7 (10.4)
RLL/LLL	4 (0.06) / 10 (14.9)
**Pathological findings**	
AAH	15
AIS	24
MIA	28

*LLL*, left lower lobe; *LUL*, left upper lobe; *RLL*, right lower lobe; *RML*, right middle lobe; *RUL*, right upper lobe.

### Image acquisition

All images were obtained using 64-detector (GE Discovery HD 750) scanners. The protocol parameters for each HRCT scanner were as follows: section width, 1.25-mm; reconstruction interval, 1.25-mm; tube voltage, 120KVp; tube current, 220 mA; beam pitch, 0.938; rotation time, 0.5s; and acquisition matrix, 512 × 512. Unenhanced spiral acquisitions were obtained with breath-hold from thoracic inlet to lung bases with images reconstructed at a slice thickness and interval of 1.25 mm. Images were reconstructed using a standard algorithm (GE bone algorithm). All images were sent to GE ADW 4.5 workstation and underwent multi-planar reconstruction (MPR) (including coronal and sagittal) with a thickness of 1.25 mm. HRCT Histogram was obtained using volume rendering (VR) method and was utilized to analyze the volume of lesions. All studies were reviewed on a PACS workstation (GE Healthcare, Milwaukee, WI) with window level −500 to −700 HU and width 1400 HU.

### Image analysis

HRCT images were analyzed jointly by 2 radiologists with 11 and 8 years of experience in thorax imaging. Both readers were blinded to the patients’ clinical data, and a consensus was reached for each patient. HRCT images obtained in same patients were assessed separately in random order, with an interval of at least 2 weeks. Data analysis was based on source images and images of MPR. The imaging characteristics and volume of pure GGNs were recorded. The pure GGN was considered as spherical when its diameter was almost the same on axial, coronal, and sagittal views [11]. GGN shape was classified as nodular (round, oval or polygonal in shape) or irregular (shape different from above all) [16]. Lesion borders were classified as smooth, notched, lobulated, spiculated. Smooth margins were clear-cut; A notched was defied as V-shaped which the border deeper than 3 mm; lobulated margins were irregular; and spiculated margins were irregular with disordered profiles [17, 18]. The relationship between blood vessels and pure GGN was classified into III types (I, vessels passing by or through pure GGN; II, intact vessels passing through pure GGN with tiny branches; III, the lesion vessels were wider, distorted or tortuous compare to other vessels at the same image).

**Fig 1:**
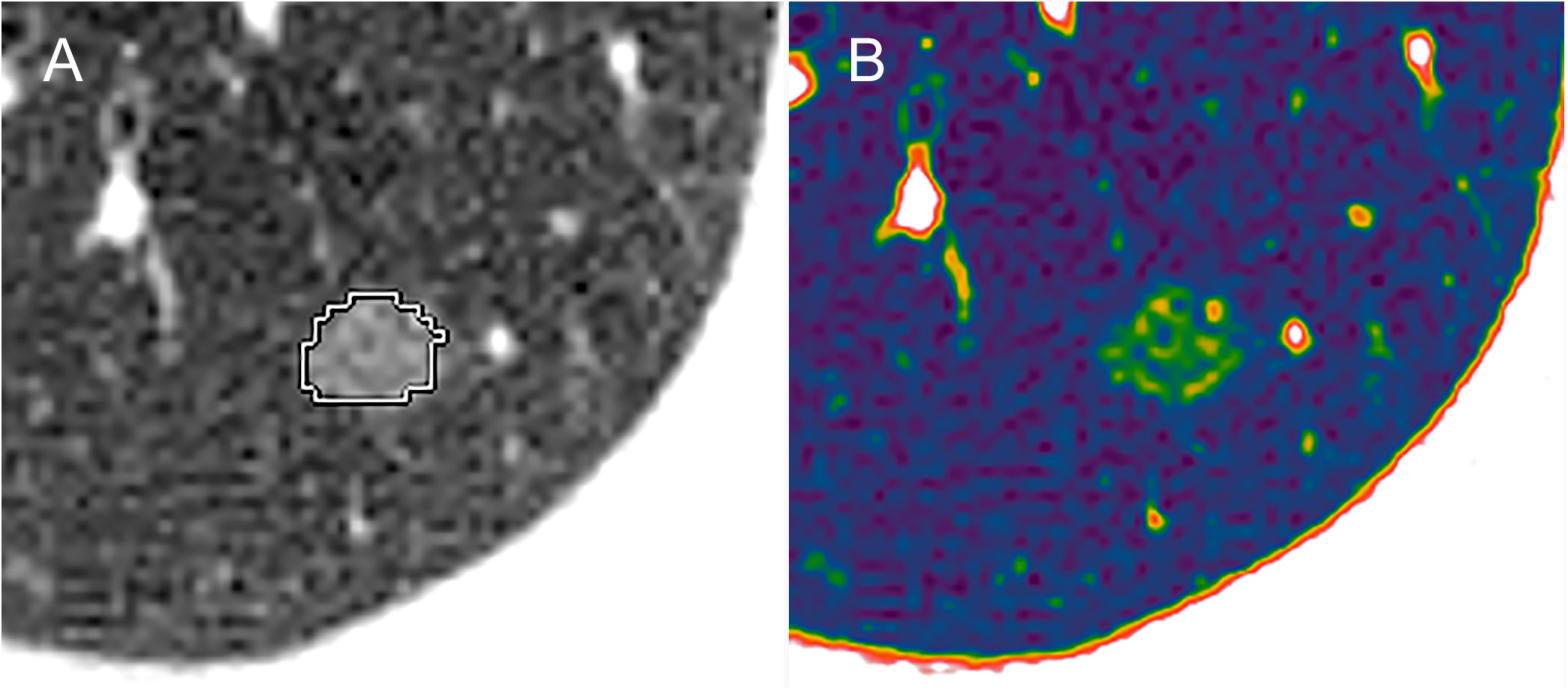
Nodule contouring process. (A) The edge of the nodule is traced automatically. (B) The segmented nodule shows different colour in the two-dimensional colour mapping.

For each pure GGN, diameter was measured on axial images (the diameter was defined as the average length of long and short diameters [19]). The mean HRCT attenuation value was measured using region-of-interest (ROI) cursors, which traced the edge of the tumor using automatic tracing juts along its internal edge, on the slices containing the region of the lesion with greatest diameter. Large vessels and pulmonary arteries were excluded from ROI [20].The volume was performed by automatically outlining the lesion perimeter on all axial images on which the pure GGN was visible using software integrated in the workstation. After the nodule segmentation, the workstation created a density histogram of the entire nodule using a volume rendering approach. Two dimensional color mapping image showed different colors in the segmented nodule, which reflected the density (Fig 1).All measurements were performed by two radiologists retrospectively. The average values of the data were used for statistical analysis. If the data differed between two radiologists by >2mm in diameter, >50HU in HRCT attenuation or >0.05cm^3^ in volume, the HRCT findings were reviewed and re-examined jointly by two radiologists.

### Statistical analysis

All data regarding of continuous variables were expressed as mean ± SD. For HRCT variables, means of values were recorded, and inter-observer variability was calculated using repeated measure data analysis for the intra-class correlation coefficient (ICC). Statistical differences between pre-invasive lesions and MIA were analyzed using independent sample *t* test for differences in diameter and HRCT attenuation. The volume data was analyzed using Mann-Whitney U test. The Fisher’s exact was used for assessment of lesion location and radiographic characteristics. For the differentiation of pre-invasive lesions from MIA, the optimal cut-off values of lesion diameter, HRCT attenuation and volume values were calculated using receiver operating characteristic (ROC) curve analysis. The optimal cut-off values were determined as the point closest to the upper left hand corner of ROC curve. Statistical analysis was performed using SPSS (version 17.0 SPSS, Chicago, III). *p* value less than 0.05 was considered as statistically significant.

## Results

### HRCT findings and volume of pure GGN

Inter-observer agreement index for diameter, mean HRCT attenuations and volume of pure GGNs was high (ICC>0.75). Intra-class correlation coefficients were 0.97 (95% confidence interval [CI]: 0.94–0.98) for size, 0.77 (95% CI: 0.68–0.81) for HRCT attenuation, and 0.79 (95% CI: 0.68–0.81) for tumor volume. Univariate analyses showed that lesion diameter, mean HRCT attenuation, and volume value differed significantly between two groups (P<0.001, P = 0.03 and P<0.001, respectively).

**Fig 2:**
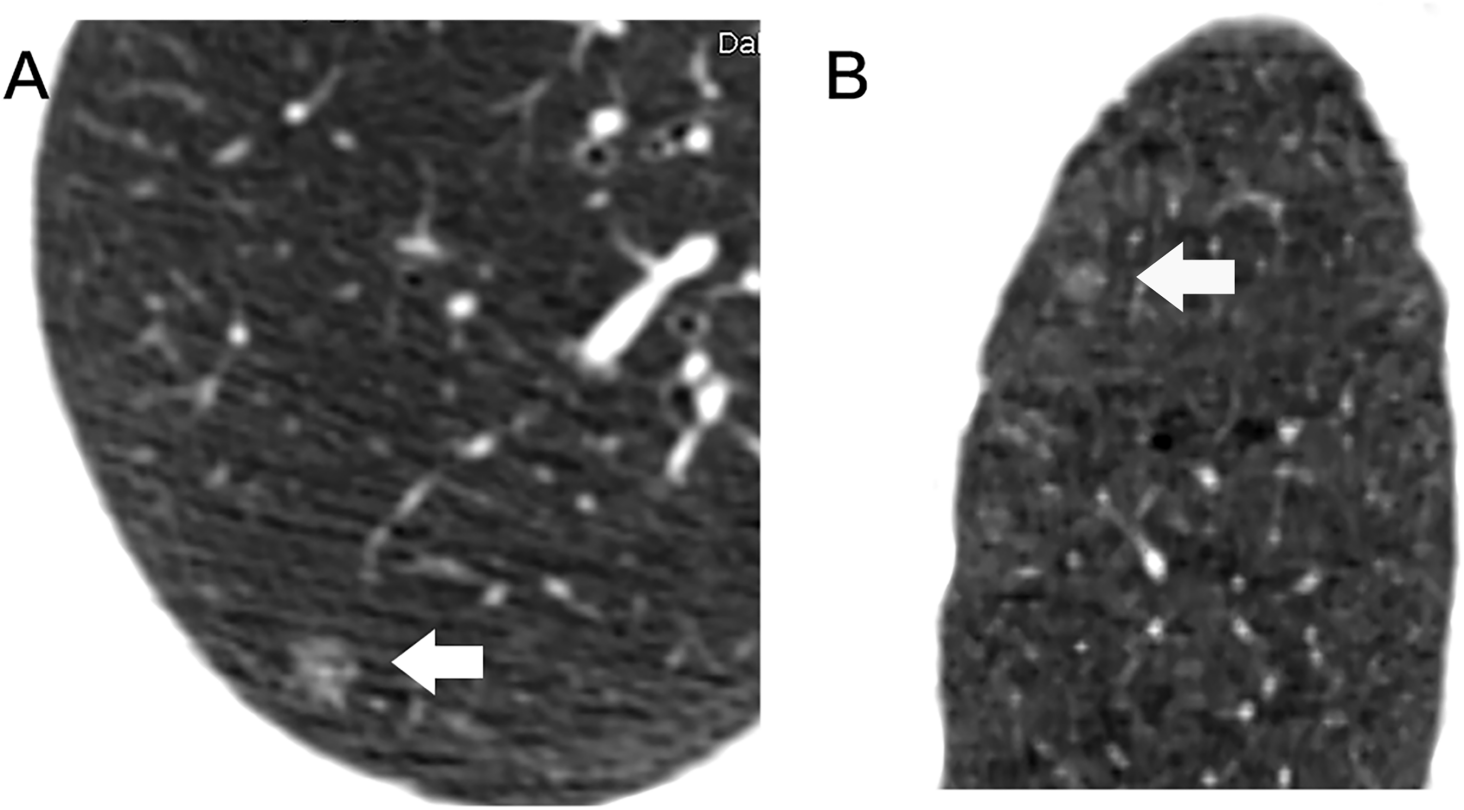
Radiologic findings of pure GGN. A 58-year-old woman with an AAH in the upper lobe of right lung. Axial (A) and coronal (B) HRCT images show a pure GGN with a round shape and a smooth border, and a vessel through pure GGN (type I) (coarse arrow).

**Fig 3:**
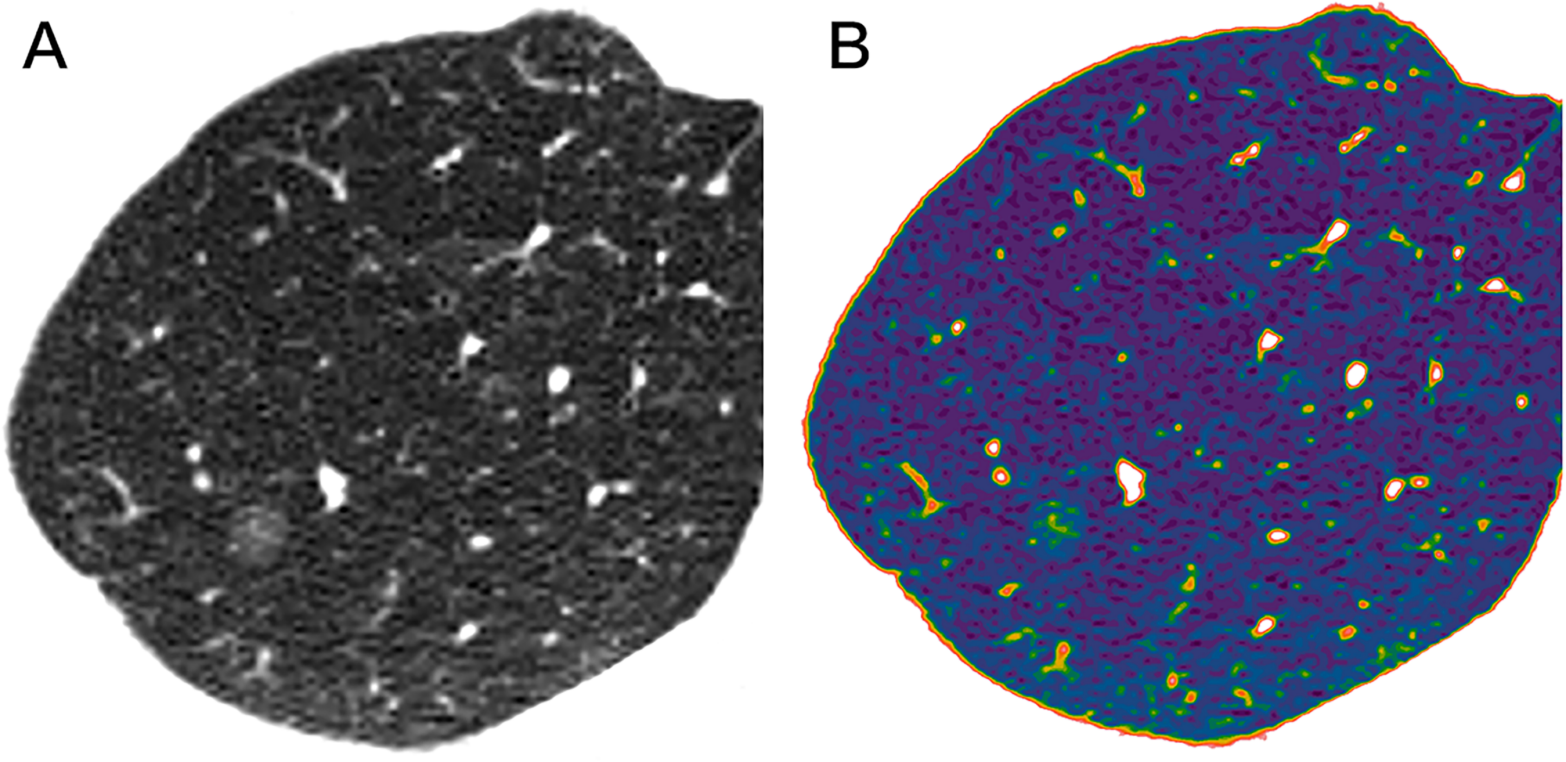
A 33-year-old woman with an AAH in the upper lobe of right lung. Axial (A) and colour mapping (B) images show a 7mm well-defined round nodule with smooth border, and vessels passing through pure GGN (coarse arrow).

**Fig 4:**
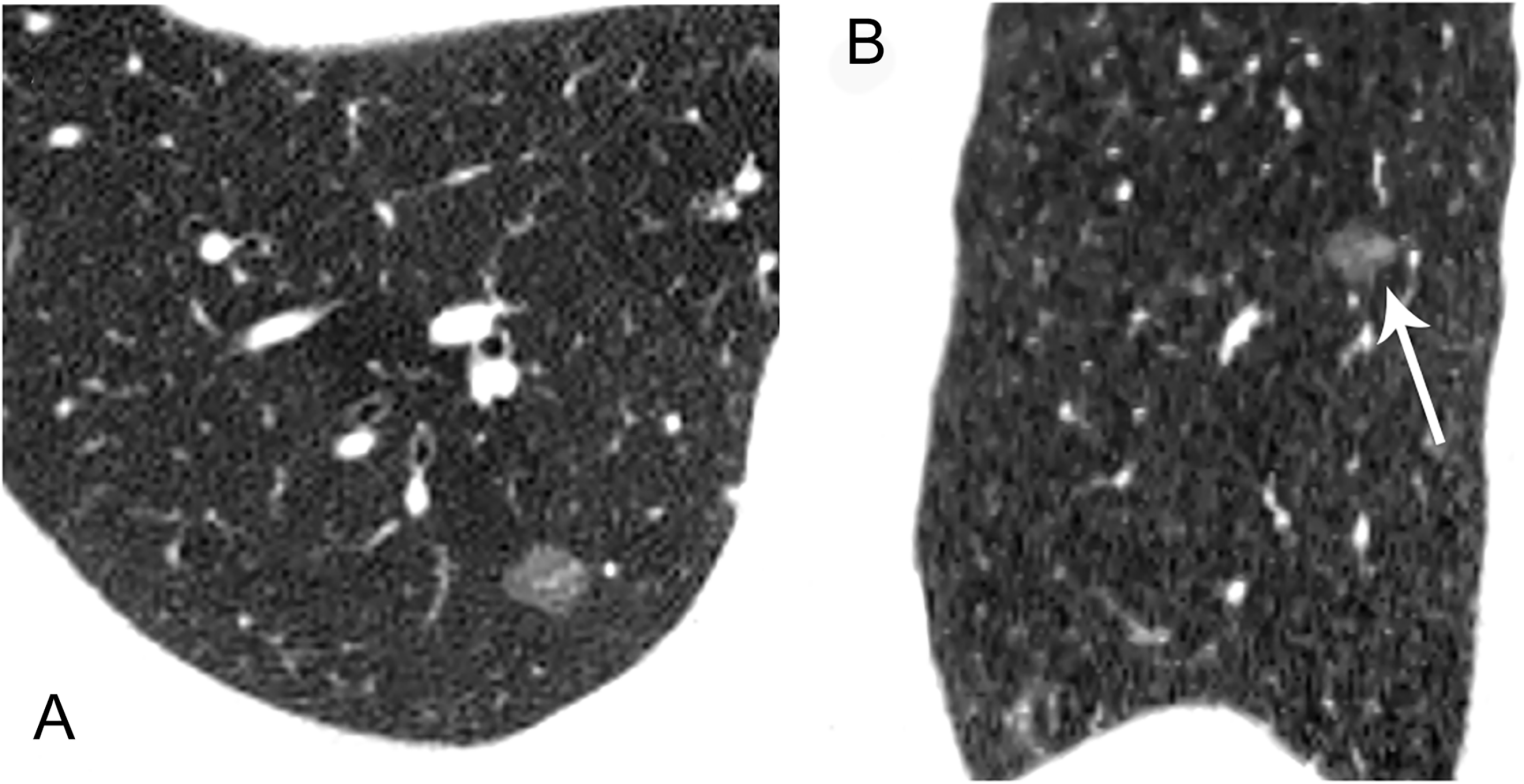
A 10mm pure GGN in axial image (A) of the lower lobe of right lung in a 62-year-old woman, sagittal (B) HRCT image shows an oval nodule with a notched border (arrow), and vessels passing through the pure GGN with tiny branches.

**Fig 5:**
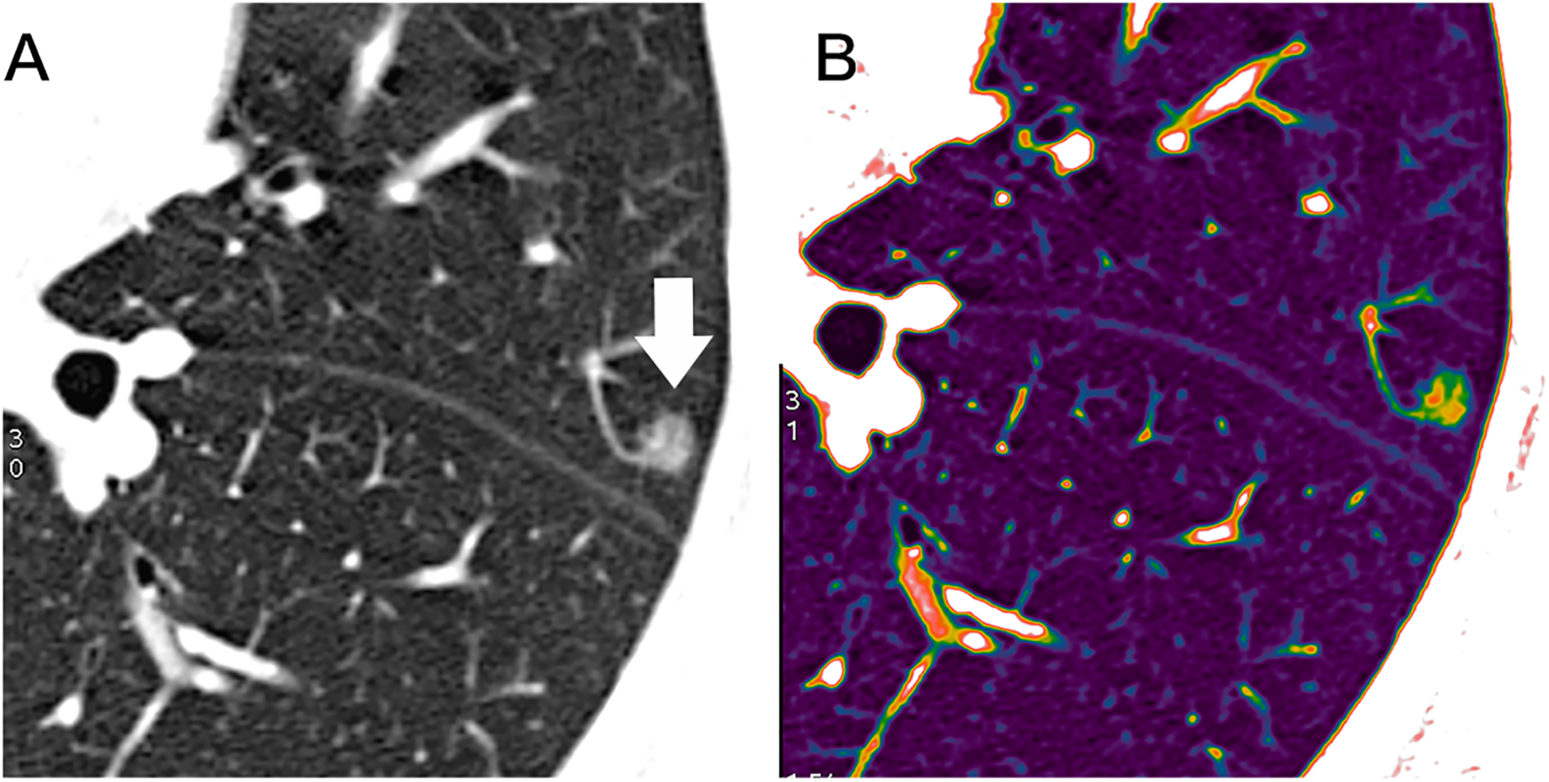
A 55-year-old woman with an AIS in the upper lobe of left lung. Axial (A) and colour mapping (B) images show a pure GGN. The 6mm nodule shows an oval shape, a smooth border, and vessels through with tiny branches (coarse arrow).

**Fig 6:**
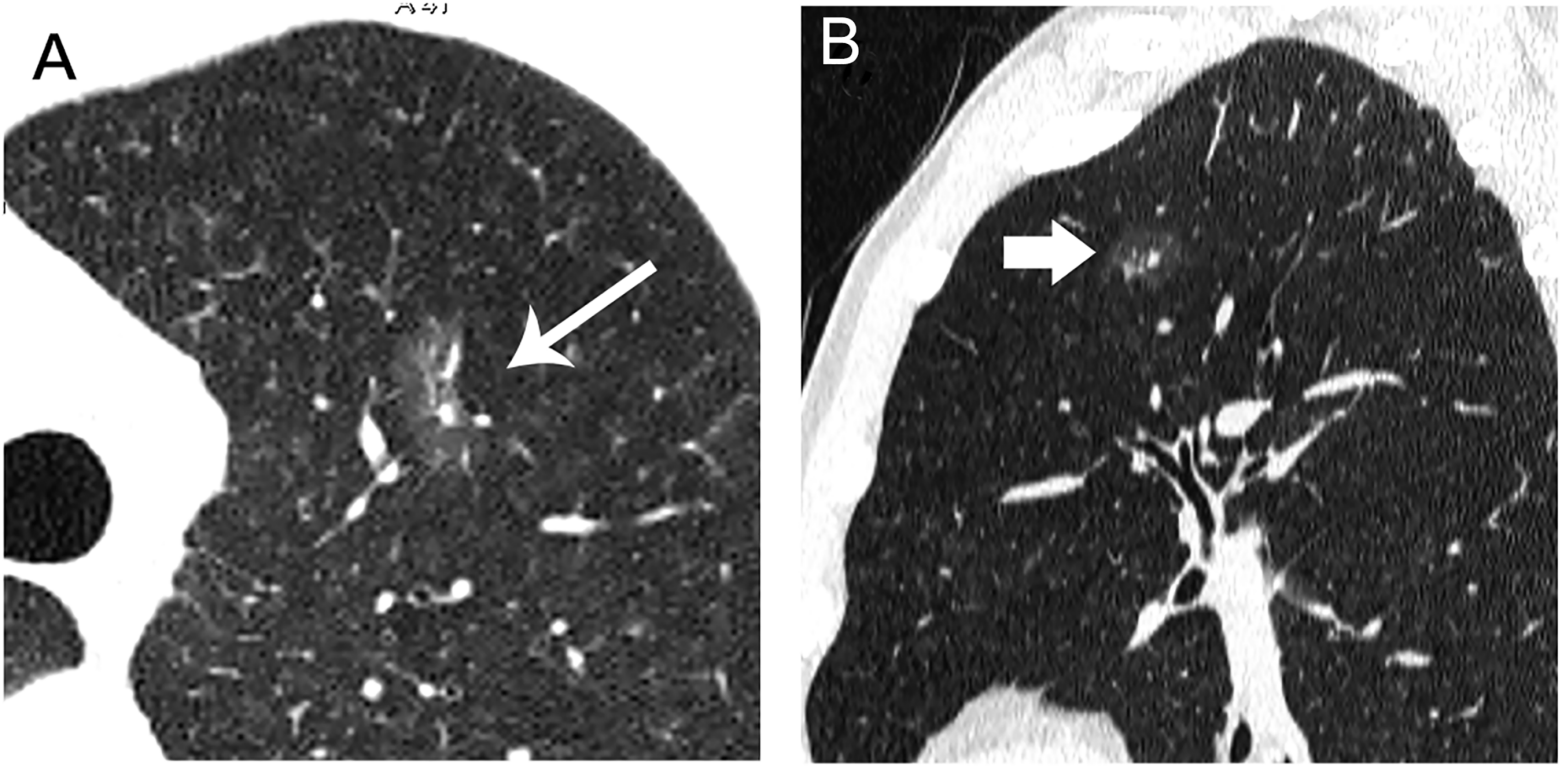
A 55-year-old man with a MIA in the upper lobe of left lung. Axial (A) and coronal (B) HRCT images show a 13.2mm irregular nodule with spiculated margin (arrow), and intact vessels through lesion with tiny branches (coarse arrow).

**Fig 7:**
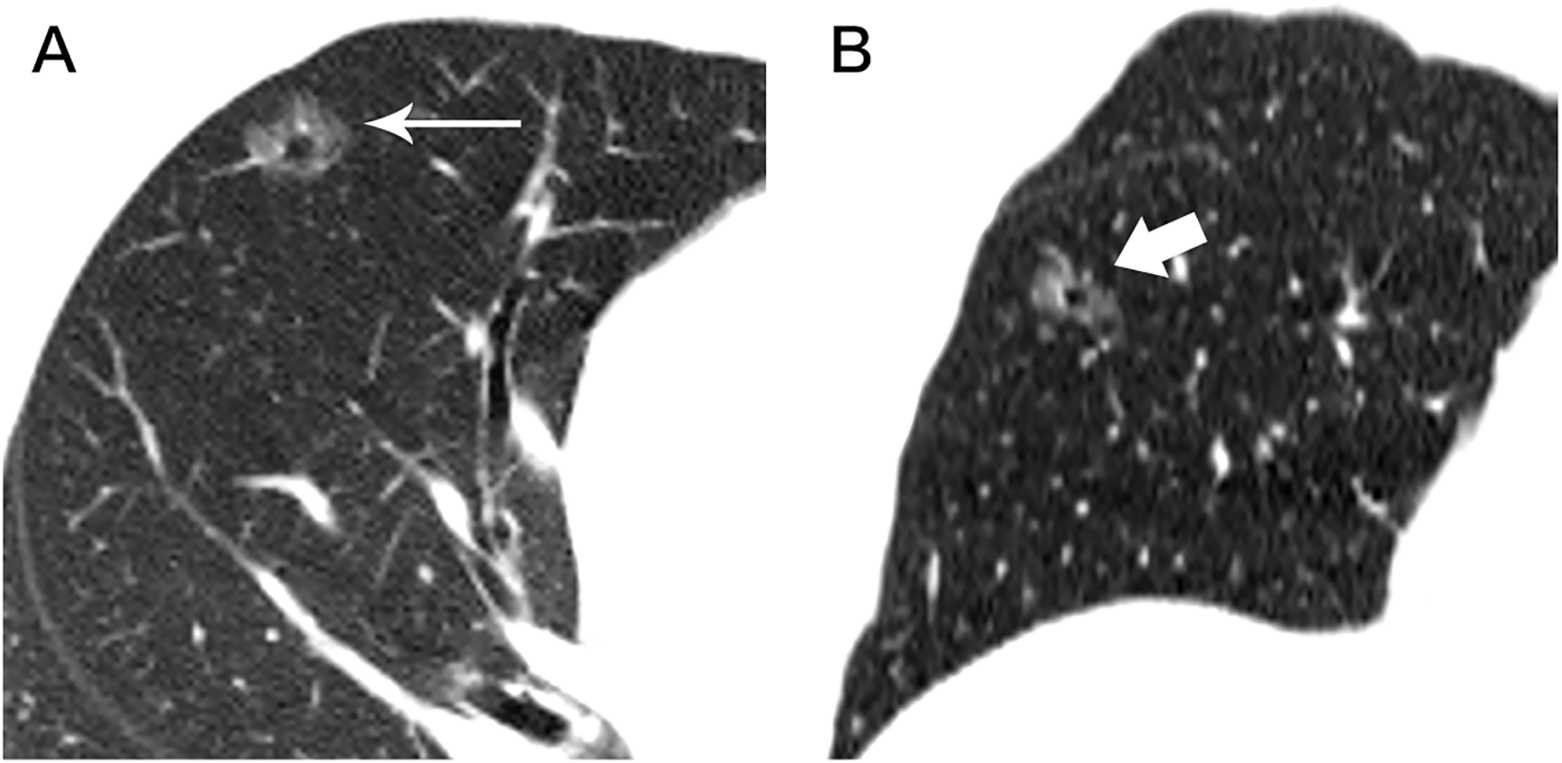
A 52-year-old man with a MIA in the middle lobe of right lung. Axial (A), sagittal (B) images show an 11.9mm nodule with polygonal shape, irregular and lobulated margin, bubbly lucencies (arrow) and intact vessels distorted (coarse arrow).

The location of lesion showed no significant difference between pre-invasive group and MIA group (F = 4.464, P = 0.34).Among HRCT findings, pre-invasive lesions appeared as round or oval (F = 13.456, P = 0.002) and frequently smooth or notched (F = 15.742, P = 0.001) in comparison to MIA (Figs 2–7).

The relationships between GGNs and vessels were analyzed based on relative position. Among of 67 GGNs, the type I, II, III relationships of pure GGNs and vessels were observed in 17, 30, 20 cases, separately (Figs 2–7). Obviously, the number of type II was the largest group with amount of 44.8%. Significant difference was shown between pre-invasive group and MIA group (F = 31.444, P<0.010). The relationships of Type II and III of pure GGNs and the characteristics of vessels passing through pure GGN strongly suggested high likelihood of malignancy (Table 2).

**Table 2:** HRCT morphologic features of lung pre-invasive lesion and MIA.

Morphologic features	Pre-invasive lesion (n = 39)	MIA (n = 28)	*p*-value^a^
**Diameter (mm)**	7.03 ± 1.74	10.16 ± 3.75	0.001^b^
**Mean HRCT value (HU)**	-656.10 ± 50.71	-606.00 ± 67.37	0.03^b^
**Volume (cm**^**3**^**)**	0.18 ± 0.09	0.69 ± 0.66	0.001^c^
**Shape**			
Round/oval	20 (51.3%) / 16 (41.0%)	7 (25%) / 8 (28.6%)	0.002
Polygonal/irregular	2 (5.1%) / 1 (2.6%)	10 (35.7%) / 3 (10.7%)	
**Border**			
Smooth/Notch	13 (33.3%) / 20 (51.3%)	7 (25%) / 8 (28.6%)	0.001
Lobulated/Spiculated	5 (12.8) / 1 (2.6)	10 (35.7) / 3 (10.7)	
**Pure GGN-vessels**			
Type I	16 (41.0%)	1 (3.6%)	0.001
Type II	21 (53.8%)	9 (32.1%)	
Type III	2 (5.1%)	18 (64.3%)	

*Pure GGN-vessels*, the relationship between pure GGN and the blood vessels.

^a^ Fisher’s exact test.

^b^ The independent sample t test.

^c^ Mann-Whitney U test.

### ROC analysis of pure GGN

**Fig 8:**
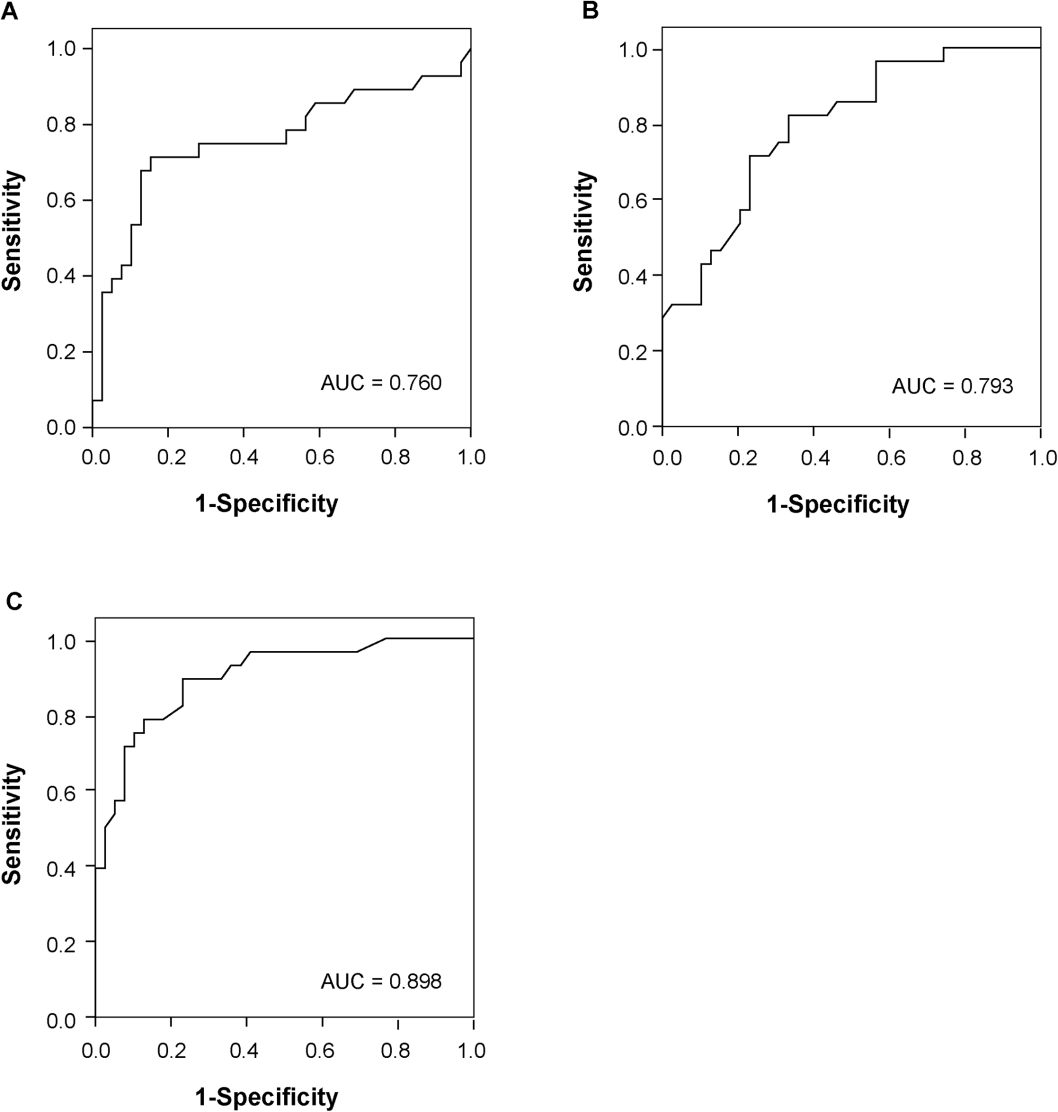
Receiver operating characteristic (ROC) curve for HRCT variables. The area under ROC curve (AUC) for lesion diameter was 0.760 (A), the AUC for mean HRCT attenuation 0.793 (B), and the AUC for lesion volume 0.898 (C). The optimal cut-off value used for lesion diameter, mean HRCT attenuation and volume to differentiate pre-invasive lesions from MIA was 8.18 mm (sensitivity, 75.0%; specificity, 71.8%; PPV, 44.8%; and NPV, 52.7%), -602 HU (sensitivity, 71.4%; specificity, 76.9%; PPV, 49.1%; and NPV, 50.6%), and 0.33 cm3 (sensitivity, 78.6%; specificity, 87.2%; PPV, 51.9%; and NPV, 48.8%), separately.

ROC analysis revealed that the area under ROC curve (AUC) for lesion diameter was 0.760, the AUC for mean HRCT attenuation 0.793, and the AUC for lesion volume 0.898. The optimal cut-off value used for lesion diameter, mean HRCT attenuation and volume to differentiate pre-invasive lesions from MIA was 8.18 mm(sensitivity, 75.0%; specificity, 71.8%; PPV, 44.8%; and NPV, 52.7%), -602 HU (sensitivity, 71.4%; specificity, 76.9%; PPV, 49.1%; and NPV, 50.6%), and 0.33 cm3 (sensitivity, 78.6%; specificity, 87.2%; PPV, 51.9%; and NPV, 48.8%), separately (Fig 8).

## Discussion

Pure GGNs detected using HRCT are commonly identified abnormalities with different pathological results. Currently, MIA and pre-invasive lesions as pure GGNs garner more attention in clinical practice.

The optimal size cut-off value for discriminating MIA from pre-invasive lesions as pure GGNs was 8.18 mm according to this research. The larger pure GGN is, the more likely to be malignant. It correlates well with a report [10], which showed that pure GGNs with size greater than 8 mm was associated with higher rate of malignancy. However, the other study [9] showed that 10 mm was the optimal cut-off value for distinguishing invasive adenocarcinoma from pre-invasive pure GGNs. With longer life expectancy and for new adenocarcinoma classification, peoples and clinicians pay much attention to health check-up, Therefore, much more pure GGNs are early detected. It may also due to the different classification. For example, some studies included invasive pulmonary adenocarcinomas as malignant pure GGN, which were not analyzed in this assortment.

Moreover, HRCT attenuation of lesions could be a useful method for distinguishing pre-invasive adenocarcinoma from MIA. High HRCT attenuation and volume values of pure GGNs reflect more tumor cells that grow along alveolar septa [21]. A study showed that the pure GGN with a mean HRCT attenuation value of ≥−670 HU was an independent risk factor for tumor growth [10]. The value of -602HU was an optimal cut-off value for differentiating pre-invasive adenocarcinoma from MIA in this study. Due to less or even none of invasive neoplastic cells growth, pre-invasive adenocarcinoma displayed lower attenuation values than MIA.

On the other hand, the optimal cut-off value of volume using to differentiate pre-invasive lesions from MIA was 0.33 cm^3^ in this study. And the ROC analysis of volume implied an AUC value of 0.898. The proposed histogram analysis of HRCT volume provides higher discriminatory ability, and aids in improved classification of pre-invasive adenocarcinoma and MIA of pure GGNs. One report also showed that pure GGO also showed rapid volume expansion if it changed to mix GGO [14].

Additionally, there are other significant HRCT morphologic characteristics of pure GGNs. The pre-invasive lesions often indicate round or oval shape, and smooth or notched margin, while MIA shows oval, polygonal or irregular shape, together with shallow notch, lobular or speculated margin. The presence of central bubbly lucencies is another feature described in adenocarcinoma [18]. Air bronchograms and bubble-like lucencies are not significantly predictive in some investigations[13,22,23], which reported the notched signs, spiculations, and pleural tags to be more common in cases of invasive adenocarcinomas. The difference of HRCT features in pure GGNs is probably due to histopathologic dissimilarity in the amount of alveolar airspace and cellular components contained within the nodule or the thickness of alveolar walls [12].

As to the relationship between pure GGNs and supplying blood vessels, it was categorized into three types in this study. AAH was defined as a small focal proliferation of mild to moderately atypical type II pneumocytes and/or clara cells lining alveolar walls. Therefore, type II is more common in AAH. The type III relationship exists more in MIA which resulted from an increased neoplastic cells. Interstitial fiber hyperplasia and malignant tissues may become more severe, in which the vessels present as tinier branches, wider or converging toward a lesion. Furthermore, tumor metabolism is faster than it in normal tissues and needs more blood supply [24]. It was found [25] that estrogen receptor alpha and beta expression distinguished a subset of NSCLC that had defined clinic-pathologic and genetic features. Therefore, the relationships between pure GGNs and supplying blood vessels may indicate whether or not this nodule needs surgical treatment.

There are some limitations which could not be overlooked. The study was retrospective, which may be affected by the bias of design. For example, Pure GGNs of benign lesions were excluded. Furthermore, discrete cutoffs may affect values for diagnosis. A small sample size was the other limitation of this study.

## Conclusions

HRCT characteristics of pure GGNs, including diameter, volume, shape, margin of lesions, mean HRCT attenuation, together with the relationship between pure GGNs and supplying blood vessels, can help to differentiate MIA from pre-invasive lesions to some extent.

## Abbreviations

AAH: adenomatous hyperplasia
AIS: adenocarcinoma in situ
GGN: pure ground-glass nodules
HRCT: high-resolution computed tomography
MIA: minimally invasive adenocarcinoma
MPR: multi-planar reconstruction
ROC: receiver operating characteristic
VR: volume rendering
